# Perspective on the Use of Sulfated Polysaccharides from Marine Organisms as a Source of New Antithrombotic Drugs

**DOI:** 10.3390/md13052770

**Published:** 2015-05-06

**Authors:** Paulo A. S. Mourão

**Affiliations:** 1 Connective Tissue Research Laboratory, University Hospital Clementino Fraga Filho, Rio de Janeiro, RJ 21941-590, Brazil; E-Mail: pmourao@hucff.ufrj.br; Tel./Fax: +55-21-3938-2090; 2 Program of Glycobiology, Institute of Medical Biochemistry, Federal University of Rio de Janeiro, Caixa Postal 68041, Rio de Janeiro, RJ 21941-590, Brazil

**Keywords:** sulfated fucans, sulfated galactans, anticoagulant drugs, glycosaminoglycan-like polysaccharides, fucosylated chondroitin sulfate, fucoidan, carrageenans

## Abstract

Thromboembolic diseases are increasing worldwide and always require anticoagulant therapy. We still need safer and more secure antithrombotic drugs than those presently available. Sulfated polysaccharides from marine organisms may constitute a new source for the development of such drugs. Investigation of these compounds usually attempts to reproduce the therapeutic effects of heparin. However, we may need to follow different routes, focusing particularly in the following aspects: (1) defining precisely the specific structures required for interaction of these sulfated polysaccharides with proteins of the coagulation system; (2) looking for alternative mechanisms of action, distinct from those of heparin; (3) identifying side effects (mostly pro-coagulant action and hypotension rather than bleeding) and preparing derivatives that retain the desired antithrombotic action but are devoid of side effects; (4) considering that sulfated polysaccharides with low anticoagulant action on *in vitro* assays may display potent effects on animal models of experimental thrombosis; and finally (5) investigating the antithrombotic effect of these sulfated polysaccharides after oral administration or preparing derivatives that may achieve this effect. If these aspects are successfully addressed, sulfated polysaccharides from marine organisms may conquer the frontier of antithrombotic therapy and open new avenues for treatment or prevention of thromboembolic diseases.

## 1. We Need New Antithrombotic Drugs

Cardiovascular diseases are the leading cause of mortality worldwide. Among them are the thromboembolic events due to the formation of a thrombus or clot inside the circulatory system. Stasis, changes in blood coagulation, damage of the vascular wall and changes in the concentrations of leukocytes or platelets cause thrombus formation.

The occurrence of thromboembolic processes necessarily requires anticoagulant therapy. Dicumarinic (or warfarin) was the first widely used anticoagulant shortly followed by heparin. Heparin has the highest negative charge density of any known biomolecule described in vertebrate tissues so far. Just behind insulin, heparin is the second-most used naturally occurring drug in medicine. Besides being one of the first biopolymers employed as a medicine, and certainly the principal example of a carbohydrate-based drug, heparin is perhaps one of the oldest natural products still in use [1]. Its clinical use dates back more than 65 years [2]. Although it is primarily destined for treatment and prophylaxis of thromboembolic disorders, extracorporeal circulations during cardiovascular surgeries [3] and hemodialysis [4] also require heparin.

Besides its long-standing and varied clinical applications, heparin long-term therapy causes a number of side effects, such as bleeding, thrombocytopenia, changes in lipid metabolism and osteoporosis [5]. Additionally, heparin originates from animal tissues, and therefore is highly susceptible to contamination by pathogenic particles, such as in the case of spongiform encephalopathy. Furthermore, there is doubt if animal sources of heparin (mostly porcine or bovine intestine) will match the increasing demand for this drug. Nowadays, EUA and European countries obtain heparin exclusively from porcine intestinal mucosa. These countries are now looking for new sources of the drug [6]. However, heparins extracted from different tissues may possess distinct structures and activities, requiring separate monographs in the Pharmacopeia. This is the case of heparins obtained from bovine and porcine intestinal mucosa [7].

Even more serious are the recent reports of contamination of heparin preparations. In late 2007, the microorganism *Serratia marcescens* contaminated unopened heparin syringes, which leaded to a recall in the USA [8]. In early 2008, there were reports of contamination of heparin preparations with oversulfated chondroitin sulfate. This contaminant has a potent hypotensive effect and induces anaphylactic reactions. These side effects were associated with the death of ~200 patients in the United States [9,10]. Another negative example related to the use of mammalian heparin, which took place in Brazil, was the increase of bleeding when porcine intestinal heparin was replaced by bovine intestinal heparin [7,11].

These observations indicate we still need to search for new antithrombotic drugs, and marine organisms may constitute new sources of heparin analogs.

## 2. A Step Forward in the Study of Sulfated Polysaccharides from Marine Organisms

**Figure 1:**
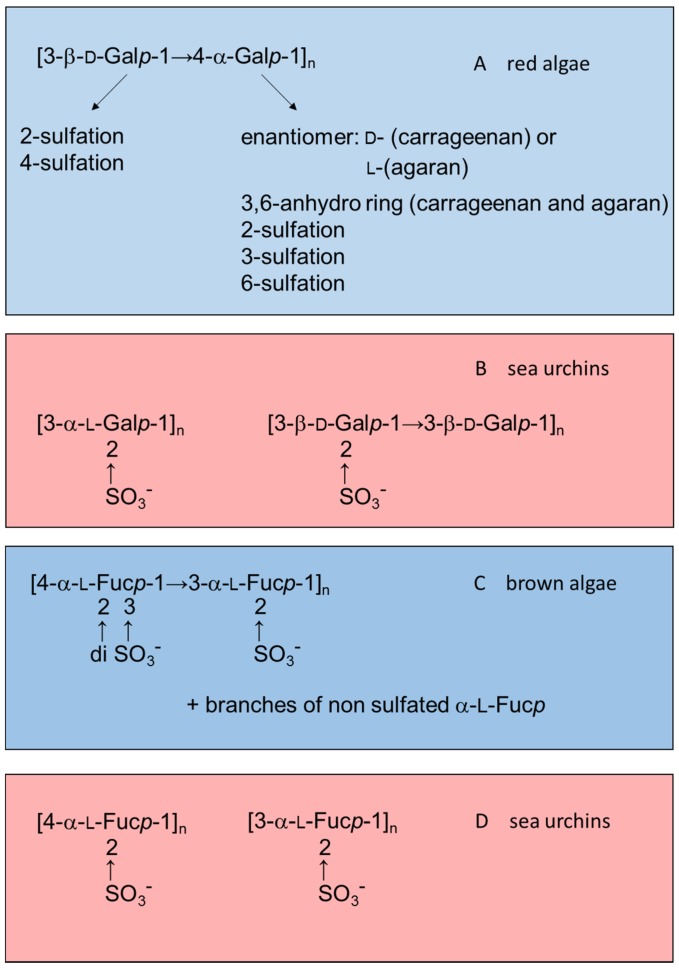
Examples of the structures of sulfated galactans from (**A**) red algae [15,16,17,18,19]; (**B**) sea urchins [20,21]; (**C**) sulfated fucan from brown algae [22,23,24]; and (**D**) from sea urchins [25,26]. Structures of the sulfated polysaccharides from marine algae highlighted in blue and those from sea urchins in red.

Marine organisms are a rich source of new substances with potential applications in medicine, though they are not yet well-explored. In particular, sulfated polysaccharides from marine invertebrates and algae possess unique structures and specific biological effects when tested in mammalian systems. The most abundant sulfated polysaccharides found in algae and marine invertebrates are sulfated fucans (also known as fucoidan when isolated from brown algae) and sulfated galactans (also known as carrageenans when isolated from red algae) [12,13,14]. In general, algal polysaccharides have more complex structures than polymers from marine invertebrates. Figure 1 illustrates this observation by comparing structures of sulfated galactans and sulfated fucans from red or brown algae (highlighted in blue) with those from sea urchins (in red).

Most studies on the effects of anticoagulant sulfated polysaccharides aim at selecting the most active native or chemically sulfated derivatives of natural polysaccharides in biological assays. Thus, the anticoagulant activity of the sulfated fucans increases with increasing sulfate content [27,28]. These studies follow an approach seeking to establish similarities between these molecules and heparin, the gold standard. The objective is to select active compounds based on general coagulation tests (mostly clotting assays). Thereafter, the aim is to compare the effects of these polysaccharides with those of heparin in more specific assays looking for similarities between them.

This approach raises skepticism since it is difficult to believe in the possibility of mimicking the effects of heparin on the coagulation system using sulfated polysaccharides with very distinct structures. The mechanism of the anticoagulant action of heparin constitutes a paradigm of interaction between carbohydrate and protein that triggers a potent biological effect [29]. A rare pentasaccharide sequence in the heparin molecule is responsible for the interaction and conformational activation of antithrombin, which is the major plasma serpin. This is the initial step, which determines the inhibition of serpin-dependent proteases of the coagulation system, especially thrombin and factor Xa.

Attempts to simulate the effect of heparin using other sulfated polysaccharides led to criticism that the structures of these compounds cannot simulate the same effect of heparin due to the obvious absence of the specific pentasaccharide sequence with high antithrombin affinity. The observation that a sulfated galactan, with high anticoagulant activity, does not induce the same conformational change on antithrombin as heparin supports this view [30]. The skeptics attributed the anticoagulant activity of the sulfated polysaccharides from marine organisms to non-specific and low-affinity interactions between their highly sulfated carbohydrate chains and basic regions in proteins of the coagulation system.

Another event which discouraged the study of sulfated polysaccharides as anticoagulants was the report that oversulfated chondroitin sulfate has an anticoagulant effect, although with much less potency than heparin and one that is associated with a strong toxic effect. This polysaccharide activates factor XII and releases bradykinin with potent hypotensive effect. Contamination of heparin preparation with oversulfated chondroitin sulfate has been responsible for ~200 deaths in the USA [9,10]. Sulfated polysaccharides from algae and marine invertebrates may have similar effects, as already reported for a sea cucumber polysaccharide [31]. These observations further increased the disbelief in the possibility of simulating the effects of heparin with other sulfated polysaccharides.

However, it is possible to follow different routes to investigate these sulfated polysaccharides from marine organisms as anticoagulant drugs using a more innovative approach. The essential point is to look for distinct effects of these sulfated polysaccharides in the coagulation system, different from those reported for heparin. Sulfated polysaccharides with new mechanisms of action may open new avenues for therapeutic applications in thromboembolic diseases. This review will focus on some of these possibilities.

## 3. The Anticoagulant Effect of Sulfated Galactans and Sulfated Fucans Depends on Specific Structures

In a study performed in our laboratory, we tested the anticoagulant effects of sulfated galactans and sulfated fucans obtained from marine invertebrates. More than 20 types of sulfated polysaccharides with well-defined and repetitive structures were tested. The substantial number of results obtained allowed us to ensure that the structure of the sulfated polysaccharide correlates with its specific effects on the coagulation system. Three examples of this type of approach are described below.

Initially we compared the anticoagulant effect of a set of sulfated fucans obtained from sea urchins, all composed of linear chains of 3-linked α-l-fucopyranosyl (Fuc*p*) units with different patterns of 2- and 4-sulfation. We observed that the presence of 2,4-disulfated Fuc*p* units is the major requirement for antithrombin-mediated anticoagulant activity [32].

**Figure 2:**
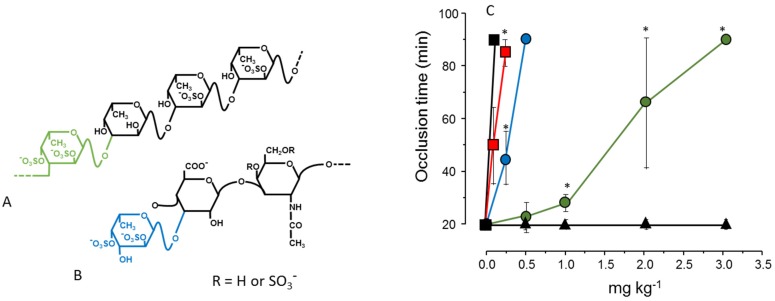
Structures (**A**,**B**) and antithrombotic effect (**C**) of sulfated polysaccharides rich in 2,4-disulfated α-l-Fuc*p* units. (**A**) The sulfated fucan is composed of [3-α-l-Fuc*p*-2,4(OSO_3_^−^)-1→3-α-l-Fuc*p*-1→3-α-l-Fuc*p*-2(OSO_3_^−^)-1→3-α-l-Fuc*p*-2(OSO_3_^−^)]*_n_* repeating units; (**B**) Fucosylated chondroitin sulfate has a chondroitin sulfate-like backbone, but contains branches of 2,4-disulfated α-l-Fuc*p* units linked to the β-d-GlcA residues on the polysaccharide core. The 2,4-disulfated α-l-Fuc*p* units in these two polysaccharides are highlighted in green or blue; (**C**) Antithrombotic effect of sulfated fucan (●), fucosylated chondroitin sulfate (●), unfractionated heparin (■), low-molecular-weight heparin (■) and vertebrate chondroitin 6-sulfate (▲) on an arterial thrombosis model induced in carotid artery of rats by laser irradiation. See Ref. [35] for further details.

In another approach we compared the antithrombotic effect of two types of polysaccharides rich in 2,4-disulfated α-l-Fuc*p* units. One of them was a fucosylated chondroitin sulfate isolated from the sea cucumber. This polysaccharide has a backbone like that of vertebrate chondroitin sulfate: [4-β-d-glucuronic acid (GlcA)-1→3-β-d-*N*-acetylgalactosamine (GalNAc)-1]*_n_* but substituted at the 3-position of the β-d-GlcA with branches of 2,4-disulfated α-l-Fuc*p* [33]. Another polysaccharide was a sulfated fucan isolated from the same invertebrate but containing 2,4-disulfated α-Fuc*p* units as part of a linear chain: [α-l-Fuc*p*-2,4(OSO_3_^−^)-1→3-α-l-Fuc*p*-1→3-α-l-Fuc*p*-2(OSO_3_^−^)-1→3-α-l-Fuc*p*-2(OSO_3_^−^)]*_n_* [34] (Figure 2, Panels A and B). The result shown in Figure 2, Panel C, illustrates the effect of these two sulfated polysaccharides in an experimental model of arterial thrombosis. Clearly, occurrence of 2,4-disulfated α-Fuc*p* as branched residues (as in fucosylated chondroitin sulfate) ensures a more potent antithrombotic effect compared to the linear polymer containing the same type of residue [35].

**Figure 3:**
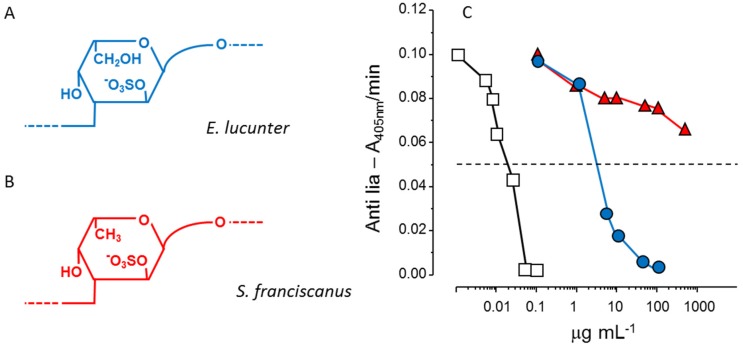
Structures and anticoagulant effect of a sulfated galactan and a sulfated fucan. (**A**,**B**) Structures of the sulfated α-l-galactan from the sea urchin *E. lucunter* (in blue, Panel A) and sulfated α-l-fucan from *S.* *franciscanus* (in red, Panel B). Both polysaccharides are 3-linked with a regular sulfation at the 2-position, but differ in their constituent monosaccharides; (**C**) Thrombin inhibition mediated by antithrombin *vs.* concentrations of the sulfated galactan (●), sulfated fucan (▲) and heparin (□). See Ref. [36] for further details.

Another example of the stereospecificity of the anticoagulant effect of sulfated polysaccharides arises from comparison between a α-l-fucan and a α-l-galactan, both 2-sulfated and 3-linked (Figure 3, Panels A and B). The sulfated α-galactan is significantly more active than the sulfated α-fucan as an antithrombin-mediated thrombin inhibitor (Figure 3, Panel C) [36].

In conclusion, pattern of sulfation, position of the residue, either internal or non-reducing unit, and type of constituent sugar [α-l-galactopyranosyl (Gal*p*) or α-l-Fuc*p* unit] ensure the anticoagulant and antithrombotic effects. The anticoagulant activity of the polysaccharide is not only a consequence of negative charge density, but of precise structural constituents. 

These studies were possible using the sulfated polysaccharides from marine invertebrates that possess well-defined structures. However, these polysaccharides occur in low concentrations, or some of the invertebrate species are scarce. In several cases, we can obtain these polysaccharides only in small amounts, which limits their practical use. A practical alternative to develop antithrombotic drug is to employ sulfated polysaccharides from marine algae, which are abundant. Nevertheless, the invertebrate polymers are model molecules and may reveal a specific structural sequence we need to look for in the algal polysaccharides.

## 4. Serpin-Independent Anticoagulant Effect

Serpins (as antithrombin and heparin cofactor II) mediate the anticoagulant activity of heparin, resulting in the inhibition of the coagulation proteases. Consequently, the effect of that glycosaminoglycan disappears on serpins-depleted plasmas. Sulfated polysaccharides from marine organisms have a similar serpin-mediated activity, demonstrated on assays using purified reagents (proteases and serpins) [16,32,33,36]. It intrigued us when it was observed that the anticoagulant effect of a fucosylated chondroitin sulfate from the sea cucumber [37] and of a sulfated galactan from red algae [38] remains unchanged when tested in serpin-depleted plasma. This observation is even more striking when tests of thrombin or factor Xa generation in serpin-depleted plasma was employed. Clearly, the inhibitory effect of the blood coagulation proteases disappears in tests using heparin, but persists with fucosylated chondroitin sulfate or sulfated galactan [37,38].

**Figure 4:**
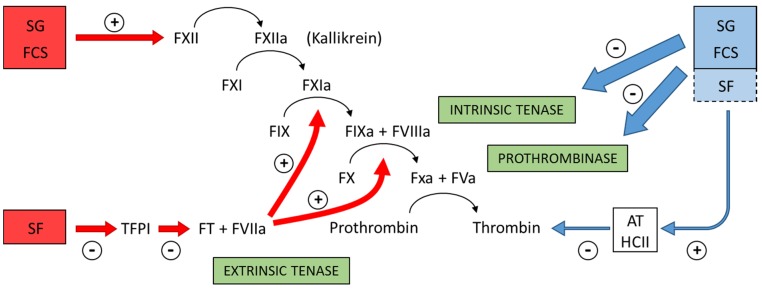
Major target sites for the sulfated polysaccharides from marine organisms on the coagulation system. Blue and red arrows indicate anticoagulant and pro-coagulant effects, respectively. + indicates activation and − indicates inhibitory effects. Anticoagulant effect: sulfated galactans (SG) from marine red algae and fucosylated chondroitin sulfates (FCS) from sea cucumbers inhibit the intrinsic tenase and prothrombinase complexes [37,38]. It is still unclear if sulfated fucans (SF) have similar effects. These polysaccharides also potentiate the inhibitory effect of antithrombin (AT) and/or heparin cofactor II (HCII) on thrombin [16,32,33,36]. Their effects on factor Xa are very modest. The serpin-independent action preponderates on the plasma system. Pro-coagulant effect: SG and FCS activate factor XII [31,39]. This effect may result in severe hypotension (due to bradykinin release) and pro-coagulant (and pro-thrombotic) action. It is unclear if SF activates factor XII. SF inhibits Tissue Factor Protease Inhibitor (TFPI), a specific inhibitor of the extrinsic tenase complex. Consequently, SF has a pro-coagulant effect [40,41].

Further examination revealed that fucosylated chondroitin sulfate and sulfated galactan inhibit the intrinsic tenase and prothrombinase complexes that are critical for the generation of factor Xa and thrombin. Assays using purified proteins of the coagulation system assure this conclusion. The sulfated polysaccharides inhibit the interaction between factor Va and factor X [38]. These results reveal an anticoagulant effect with a distinct mechanism of action compared with heparin or any other known antithrombotic drug. Figure 4 summarizes the preponderant target sites for the sulfated polysaccharides from marine organisms on the coagulation system. Blue arrows at right of the Figure indicate the anticoagulant effects.

Of course, further studies are necessary to investigate whether this distinct mechanism of action may confer favorable effects to the marine polysaccharides for the prevention and treatment of thromboembolic events. In particular, it is necessary to clarify which one of the two mechanisms (serpin-dependent or serpin-independent) is more favorable for an antithrombotic therapy.

## 5. Sulfated Polysaccharides with Low Anticoagulant Activity May Have Potent Antithrombotic Effects

Screening for new anticoagulant polysaccharides usually requires an initial assessment with general coagulation tests. The compound, which is most active, moves to a more detailed stage of evaluation using animal testing. Following this methodological guideline, our laboratory carried out a study of sulfated polysaccharides from 50 species of marine algae. The work leaded to a sulfated galactan from the red alga *Botryocladia occidentalis* with very high anticoagulant activity [16]. This polysaccharide has a relatively simple structure as compared with that of similar compounds obtained from seaweeds. The presence of 2,3-disulfated α-Gal*p* units was associated with the high anticoagulant activity of the sulfated polysaccharide.

However, we can proceed in a different way, looking for sulfated polysaccharides with low anticoagulant activity. We can propose, conceptually, that a polysaccharide with low activity in coagulation tests using *in vitro* assays may exhibit a potent antithrombotic effect when tested in animal models of thrombosis.

We followed this approach and ended up with a sulfated galactan from the red alga *Acanthophora muscoides* with low anticoagulant activity. Despite this data, we continued to go deeper with the coagulation study. The polysaccharide presented a very complex structure. Its backbone consists of alternating units of α- and β-Gal*p* units, as is the characteristic of seaweed galactans. However, we observed a wide variety of structural modifications, such as sulfation in different positions, the presence of methyl ether, and anhydrous sugars. Another unique feature of this sulfated galactan is its low molecular weight (~20 kDa) [19]. Most seaweed-sulfated galactans have extremely high molecular weights.

**Figure 5:**
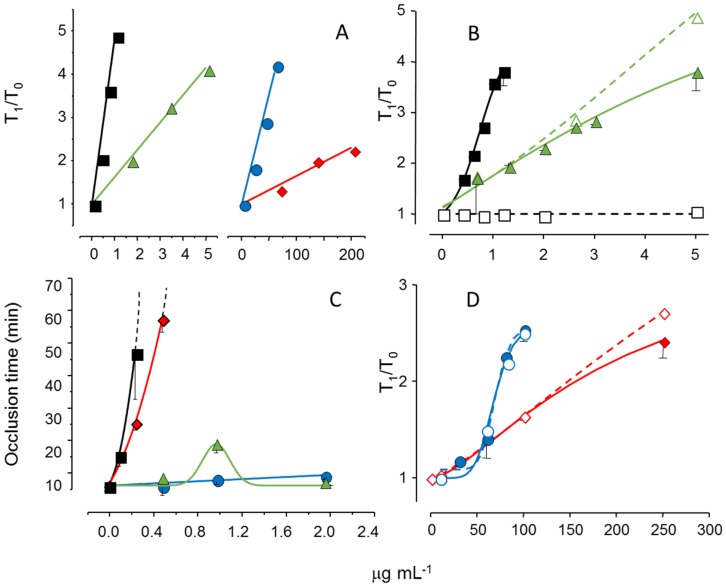
Effect of sulfated galactans on anticoagulant assays (**A**,**C**,**D**) and on arterial experimental thrombosis in rats (**B**). Different concentrations or doses of heparin (■,□), native sulfated galactan from *B. occidentalis* (▲,∆) or from *A. muscoides* (♦,◊) or the sulfated galactan from *B. occidentalis* with reduced molecular weight (●,○) were tested. In Panels C and D closed and open symbols refer to assays performed using normal or serpin-depleted plasma, respectively. Data from Ref. [19].

We reduced the molecular weight of the sulfated galactan from *B. occidentalis* using mild acid hydrolysis and obtained a derivative with the same molecular weight as that of the native polysaccharide from *A. muscoides*. The clotting assay using these two sulfated galactans, with similar molecular weights, revealed that the compound from *B. occidentalis* was more active (Figure 5, Pane A, closed circles in blue *vs.* closed diamond in red). However, more importantly, it turns out that these two sulfated galactans, with low molecular weights, are practically inactive in coagulation assays using purified coagulation proteases and serpins [19]. That is, its anticoagulant effect, albeit modest as compared to heparin, is independent of serpins. The clotting assays using serpin-depleted plasma confirmed this proposition (Figure 5, Panels B and D, compare closed *vs.* open symbols).

The next step was to test these sulfated galactans, with low anticoagulant activity and serpin-free effects, in animal models of experimental thrombosis. Figure 5, Panel C, shows the test using an experimental model of arterial thrombosis. Surprisingly, the sulfated galactan from *A. muscoides* is very active, whereas that obtained from *B. occidentalis* is completely inactive (closed circles in blue *vs.* closed diamond in red).

These results are very important because they reveal a sulfated polysaccharide, with only modest anticoagulant action on *in vitro* assay but potent arterial antithrombotic effect in the experimental animal model. We attribute this effect to the inhibition of the tenase and prothrombinase complexes that generate the coagulation proteases. This approach consolidates the molecular basis of a new antithrombotic mechanism by a sulfated polysaccharide, involving inhibition of factor Xa and thrombin generations by the tenase and prothrombinase systems.

## 6. Balance between Pro- and Anticoagulant Effects

**Figure 6:**
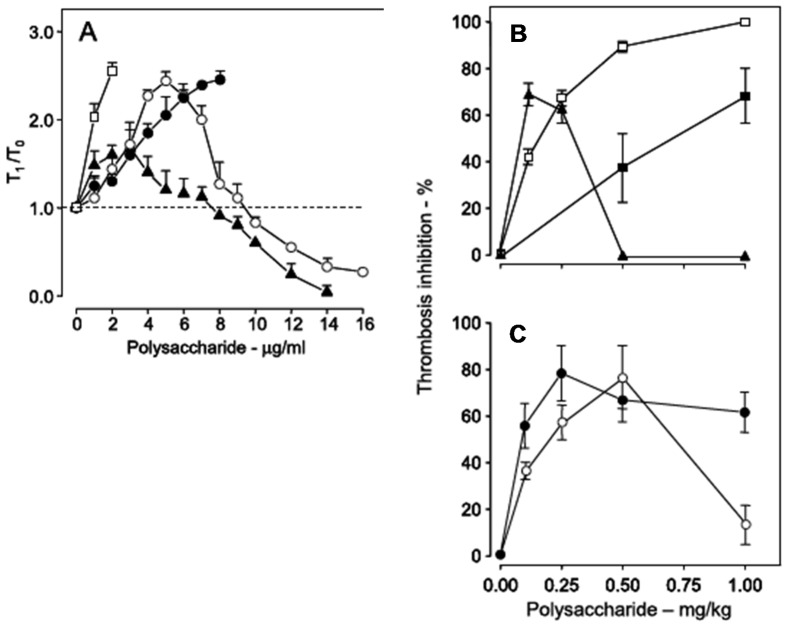
Anticoagulant activity based on recalcification time (**A**) and antithrombotic effect on a stasis thrombosis model in vena cava of rats (**B**,**C**). Different concentrations (**A**) or doses (**B**,**C**) of unfractionated heparin (□), low-molecular-weight heparin (■), native sulfated galactan (▲), ~14 kDa (O) and ~5 kDa (●) fragments were tested. In Panel A the anticoagulant activity was expressed as T_1_/T_0_, which is the ratio between the clotting time in the presence or absence of sulfated polysaccharide. The broken line (T_1_/T_0_ = 1) indicates no effect from the polysaccharide on coagulation. Data from Ref. [39].

Sulfated galactans have no bleeding tendency [19,39,42], which is the major side effect of heparin and other antithrombotic drugs. Instead, the major side effect of these polysaccharides is activation of factor XII. This action results in the release of bradykinin, leading to severe hypotension [9,10,31]. Conceptually, we expect that activation of factor XII should trigger coagulation, resulting in pro-thrombotic action. In the case of the sulfated galactan from the red alga *B. occidentalis* we observed a dual effect, as a potent anticoagulant and as an activator of factor XII [39]. This observation is very clear with the use of a recalcification time assay, without the addition of phospholipids (Figure 6, Panel A). Heparin has a potent anticoagulant action (open squares), while the sulfated galactan has a dual effect: up to ~8 μg_·_mL^−1^, an anticoagulant effect, and above 8 μg_·_mL^−1^, a pro-coagulant effect (closed triangles in Figure 6, Panel A). A 14 kDa fragment shows the same effect as the native polysaccharide but at different concentrations (open circles). Decrease of the molecular weight to ~5 kDa eliminates this dual effect (closed circles).

Because of the dual effect on coagulation, this sulfated galactan has two distinct effects in the experimental thrombosis model. At lower doses, up to approximately 0.25 mg_·_kg^−1^ body weight, it is a potent antithrombotic, similar to heparin; at higher doses, above 0.25 mg_·_kg^−1^ body weight, this effect disappears and is replaced by a pro-coagulant effect (Figure 6, Panel B), due to activation of factor XII.

In order to develop an antithrombotic drug for practical use, it is essential to dissociate the anticoagulant action from the undesirable pro-coagulant effect. We achieved this goal by means of partial acid hydrolysis of the polysaccharide. The sulfated galactan with low molecular weight (~5 kDa) proved to be a promising antithrombotic, devoid of pro-coagulant action (Figure 6, Panel C, closed circles) [39].

Curiously, even fucoidan presents the concomitant anti- and pro-coagulant effects [40,41]. Selected modified derivatives from this polysaccharide, devoid of anticoagulant activity, have been used as a therapeutic option in bleeding disorders, such as hemophilia. The pro-coagulant effect of fucoidan is distinct from the activation of factor XII proposed for the sulfated galactans. It involves a blockade of the extrinsic pathway down-regulator, the tissue factor pathway inhibitor, by the non-anticoagulant fucoidan derivatives. Red arrows at left of Figure 4 indicate the pro-coagulant effects of the sulfated polysaccharides from marine organisms.

Protamine neutralizes the effects of sulfated polysaccharides from marine organisms on the coagulation system, as occurs with heparin. It decreases the pro-coagulant effect of a sulfated galactan from red alga [42] and the anticoagulant action of fucosylated chondroitin sulfate from sea cucumber. These observations are relevant to ensure we can antagonize the effects of the polysaccharides if severe side effects occur. Induction of thrombocytopenia with the sulfated polysaccharides from marine organisms is another important aspect that requires future investigation.

## 7. Sulfated Polysaccharide with Oral Antithrombotic Effects

The use of sulfated polysaccharides as antithrombotic drug requires intravascular or subcutaneous administration, which limits their use. The new oral antithrombotic agents, which directly inhibit thrombin or factor Xa, have overcome this limitation although they may cause bleeding and have unpredicted responses [43,44].

Sulfated polysaccharides are not promising for oral administration due to their high molecular weight. Even attempts to administer heparin orally were also unsuccessful. Possibly, degradation of heparin by bacteria of the intestinal flora or by enzymes produced by the vertebrate also reduced its absorption after oral administration. However, the different chemical nature of the sulfated galactans and sulfated fucans from marine organisms makes them resistant to degradation by most enzymes that normally act on vertebrate glycosaminoglycans.

A number of studies indicate biological effects after oral ingestion of sulfated fucans from brown algae (fucoidan) [45]. Early work on digestion of these polysaccharides suggested that they were not changed by human bacterial flora and were wholly excreted [46,47]. Following oral administration to rats, we observed that fucosylated chondroitin sulfate has anticoagulant and antithrombotic effects, with low hemorrhagic action [48]. This finding allowed us to overcome the main limitation to the development of new antithrombotics based on sulfated polysaccharides. Of course, we need to extend this kind of study to other types of sulfated polysaccharides from marine organisms, especially sulfated galactans and sulfated fucans.

The observation that a sulfated polysaccharide has antithrombotic effect after oral administration raises a challenging question: how extensive is the gastrointestinal absorption of this polysaccharide? Answering this question requires a careful investigation of the metabolism of sulfated polysaccharides from marine organisms after administration to mammals, including their route of absorption and pharmacokinetics. Studies about the intestinal absorption of fucoidan have been reported recently [49].

## 8. Conclusions

In this review, we discussed possible ways to proceed in order to develop new antithrombotic drugs based on sulfated polysaccharides from marine organisms. These compounds occur in high amounts in marine algae. However, their therapeutic use in humans faces several challenges. We need to look for alternative approaches in order to establish a new paradigm, instead of continuing to look at them as “heparin-like molecules.” Possibly, we need to consider the following points:

(1) Precisely define the specific structures required for the interaction of sulfated polysaccharides with the proteins and complexes of the coagulation system.

(2) Look for alternative mechanisms of action distinct from those of heparin, including serpin-independent effects. Clarification of the precise target molecules of these sulfated polysaccharides will help to establish new drugs on a more rational basis.

(3) Identify major side effects in order to design the preparation of derivatives that retain the desirable antithrombotic action but are devoid of toxic ones. Pro-coagulant effect and hypotension, both due to activation of factor XII, instead of bleeding, may be the major obstacle for the therapeutic use of sulfated polysaccharides from marine organisms.

(4) Consider that sulfated polysaccharides with low activity on *in vitro* coagulation tests may exhibit potent activity on animal models of experimental thrombosis.

(5) Investigate the effect of these sulfated polysaccharides after oral administration. If unsuccessful, we may attempt to prepare derivatives that achieve this effect. The discovery of new oral anticoagulants derived from the sulfated polysaccharides of marine organisms will put these compounds on the frontier of antithrombotic therapeutics.
